# Deep learning-based diagnosis of disease activity in patients with Graves’ orbitopathy using orbital SPECT/CT

**DOI:** 10.1007/s00259-023-06312-2

**Published:** 2023-07-03

**Authors:** Ni Yao, Longxi Li, Zhengyuan Gao, Chen Zhao, Yanting Li, Chuang Han, Jiaofen Nan, Zelin Zhu, Yi Xiao, Fubao Zhu, Min Zhao, Weihua Zhou

**Affiliations:** 1 School of Computer and Communication Engineering, Zhengzhou University of Light Industry, Zhengzhou, 450002 Henan China; 2 School of Biomedical Engineering, Shenzhen University Medical School, Shenzhen University, Shenzhen, 518060 China; 3 Department of Applied Computing, Michigan Technological University, Houghton, MI USA; 4 Department of Nuclear Medicine, Xiangya Hospital, Central South University, Changsha, China; 5 Department of Nuclear Medicine, The Third Xiangya Hospital, Central South University, No. 138, Tongzipo Road, Changsha, 410013 Hunan Province China; 6 Center for Biocomputing and Digital Health, Institute of Computing and Cybersystems, and Health Research Institute, Michigan Technological University, Houghton, MI USA

**Keywords:** Graves’ orbitopathy, SPECT/CT, Segmentation, Classification

## Abstract

**Purpose:**

Orbital [^99m^Tc]TcDTPA orbital single-photon emission computed tomography (SPECT)/CT is an important method for assessing inflammatory activity in patients with Graves’ orbitopathy (GO). However, interpreting the results requires substantial physician workload. We aim to propose an automated method called GO-Net to detect inflammatory activity in patients with GO.

**Materials and methods:**

GO-Net had two stages: (1) a semantic V-Net segmentation network (SV-Net) that extracts extraocular muscles (EOMs) in orbital CT images and (2) a convolutional neural network (CNN) that uses SPECT/CT images and the segmentation results to classify inflammatory activity. A total of 956 eyes from 478 patients with GO (active: 475; inactive: 481) at Xiangya Hospital of Central South University were investigated. For the segmentation task, five-fold cross-validation with 194 eyes was used for training and internal validation. For the classification task, 80% of the eye data were used for training and internal fivefold cross-validation, and the remaining 20% of the eye data were used for testing. The EOM regions of interest (ROIs) were manually drawn by two readers and reviewed by an experienced physician as ground truth for segmentation GO activity was diagnosed according to clinical activity scores (CASs) and the SPECT/CT images. Furthermore, results are interpreted and visualized using gradient-weighted class activation mapping (Grad-CAM).

**Results:**

The GO-Net model combining CT, SPECT, and EOM masks achieved a sensitivity of 84.63%, a specificity of 83.87%, and an area under the receiver operating curve (AUC) of 0.89 (*p* < 0.01) on the test set for distinguishing active and inactive GO. Compared with the CT-only model, the GO-Net model showed superior diagnostic performance. Moreover, Grad-CAM demonstrated that the GO-Net model placed focus on the GO-active regions. For EOM segmentation, our segmentation model achieved a mean intersection over union (IOU) of 0.82.

**Conclusion:**

The proposed Go-Net model accurately detected GO activity and has great potential in the diagnosis of GO.

**Supplementary Information:**

The online version contains supplementary material available at 10.1007/s00259-023-06312-2.

## Introduction

Graves’ orbitopathy (GO), which is also known as thyroid eye disease or thyroid-associated orbitopathy, is the most common orbital disease in adults [1]. It is generally accepted that immune-mediated inflammation is the primary pathogenesis of GO, which is characterized by extraocular muscle (EOM) thickening, congestion, and orbital fat edema [2, 3]. Most patients with GO experience an active (dynamic) phase followed by an inactive (static) phase. For patients with mild GO, supportive treatments such as lubricant eye drops are sufficient. However, patients with severe GO may require glucocorticoids or radiation therapy to minimize inflammatory sequelae. Moreover, surgical rehabilitation is mainly indicated during the inactive phase. Therefore, an accurate method for assessing the inflammatory activity stage is important to develop efficient and precise treatments for GO patients.

The clinical activity score (CAS) is the most widely used metric to assess the activity stage in clinical settings. However, the CAS is subjective and largely depends on the opinion of the ophthalmologist. Orbital computed tomography is a fast and affordable imaging method for orbital diseases that provides accurate morphological information, such as changes in the extracted EOMs. This information can help in GO diagnoses; however, it is difficult to accurately assess the inflammatory activity stage of GO. Radionuclide imaging, such as orbital single-photon emission computed tomography (SPECT) with ^99m^technetium(^99m^Tc)-labeled diethylene triamine pentaacetic acid (DTPA), has been reported as a useful biomarker for diagnosing and staging GO activity due to its low costs, simplicity, and accuracy [4]. SPECT/CT is a hybrid modality consisting of SPECT for functional images and CT for anatomical images, thereby increasing the diagnostic accuracy [5, 6]. We recently reported that hybrid diagnostic CT and SPECT imaging has potential in assessing inflammatory activity through semiquantitative image analyses of EOMs in patients with GO [7]. Moreover, determining the DTPA uptake of EOMs through SPECT/CT might be superior to applying the CAS to predict treatment responses to periocular glucocorticoid therapy in GO patients, even if the CAS is below 3 points [5, 7].

Recently, machine learning (ML) has been widely used in the field of medical imaging, including eye imaging [8–10]. We have previously developed deep neural networks for medical image segmentation and classification [11–13]. Recently, a new segmentation method known as SV-Net was developed by our group for automatic segmentation of EOMs based on orbital CT images. The results had good agreement with the ground truth (all *R* > 0.98, *P* < 0.0001) [14]. Song et al. [15] built an ML-based model for screening GO patients using orbital CT, and Chen et al. [10] proposed a deep learning model for detecting the activity stage of GO patients through orbital magnetic resonance imaging (MRI). However, the automation level and accuracy of these approaches need to be improved.

This study is aimed at developing a two-stage automated approach to detect GO disease activity by using deep learning algorithms, including a segmentation model to extract EOMs and a classification model to distinguish active and inactive GO patients.

## Materials and methods

### Study cohort

This study included 478 patients with GO (181 males, 297 females, aged 8–82 years, mean age: 43 ± 12 years) that were referred for orbital SPECT/CT at Xiangya Hospital of Central South University between January 2017 and December 2019. Clinical information was collected from each patient, including age, sex, and ill assessment by three experienced ophthalmologists. Given the retrospective nature of the present study, the GO diagnostic criteria were based on the 2021 European Group on Graves’ Ophthalmopathy (EUGOGO) guidelines [16]. The exclusion criteria were defined as follows: (1) pregnant or lactating women, (2) other orbital inflammation, and (3) poor image quality due to avid DPTA uptake of adjacent pansinusitis. This study was approved by the Ethics Committee of Xiangya Hospital of Central South University (No. 202101021), and the requirement to obtain written informed consent was waived.

### [^99m^Tc]TcDTPA SPECT/CT scan acquisition

After intravenous administration of [^99m^Tc]TcDTPA (Chinese Atomic Energy Institute, Beijing, China) 555 MBq (15 mCi) for 20 min, orbital SPECT/CT was performed using a 16-slice SPECT/CT scanner (Preference 16, SPECT/CT, Philips Medical Systems, The Netherlands) as described in our previous report [7]. Briefly, subjects were stabilized in a supine position and asked to keep their eyes closed and stationary throughout the scan to reduce eye movements [7]. The CT scans (140 kV, 100 mA, 1 mm slice thickness, 1:1 pitch ratio) were performed first. SPECT tomographic images were then acquired at the same position by a low-energy and high-resolution collimator with 1 × magnification and a 64 × 64 acquisition matrix at 25 frames/s, with 32 projections per camera head.

### Annotations for EOM contours and GO activity

For the segmentation task, 20% of the CT image data (194 eyes) were randomly selected to generate ground truth labels for model training and validation (4:1 ratio) using fivefold cross-validation. The remaining 80% of the data (762 eyes) without ground truth labels were used as an independent test set. Two readers (LL and ZG) were trained by an senior nuclear medicine physician (MZ) to manually draw the regions of interest (ROIs) of the superior, inferior, medial, and lateral rectus on both eyes using the open annotation tool LabelMe, as previously described [14]. Thereafter, all EOM ROIs drawn by the two readers were reviewed and corrected by the senior physician in order to assure consistency and accuracy. The final segmentation results served as the ground truth labels for training the segmentation model. Additionally, a binary map derived from the all EOM ROIs was generated and served as EOM mask, as depicted in Supplementary Fig. 1. It is computed by the following equation:

1 $$\text{Binary image}\left(x,y\right)=\left\{\begin{array}{ccc}1& {\text{,}}& \text{if }\left(x,y\right) \, \in \, {\text{contour}}\\ 0& {\text{,}}& {\text{otherwise}}\end{array}\right.$$

In Equation (1), binary image (*x*, *y*) represents the pixel value at coordinates (*x*, *y*) in the binary map. If the coordinates (*x*, *y*) are within the EOM region, the corresponding pixel value is set to 1, indicating the presence of EOMs. Conversely, if the coordinates are outside the EOM region, the pixel value is set to 0, indicating the absence of EOMs.

For the classification task, the activity stage was determined based on the following criteria: (1) based on the 2021 EUGOGO guidelines [16], eyes with CASs ≥ 3/7 were annotated as active; (2) for eyes with CASs < 3/7, high [^99m^Tc]TcDTPA uptake observed in orbit was annotated as active, while the absence of [^99m^Tc]TcDTPA uptake was annotated as inactive. After the images were annotated, 475 eyes [median CAS: 3, interquartile range (IQR): 2 to 4] were labeled “active” and 481 eyes (median CAS: 1, IQR: 1 to 2) were labeled “inactive.” The test dataset was generated by randomly selecting 20% of the entire dataset, and the remaining 80% of the data were divided into training and validation sets (4:1 ratio) to perform fivefold cross-validation.

### Data preprocessing

The SPECT and CT scans were both resampled with three spline interpolations to obtain uniform 1 × 1 × 1 mm^3^ voxel images. The pixel values of the CT and SPECT images were normalized to the range of 0 to 1 to reduce inconsistencies between the two images [17–19].

A trained radiologist identified slices containing the complete orbit according to the coronal planes. Two 256 × 256 regions of interest (ROIs) centered on the optic nerves of the right, and left eyes were automatically cut from these slices. In order to increase the size of the deep-learning dataset for augmentation, all images of the left eye were flipped horizontally to maintain consistency in the orientation of the four extraocular muscles on both eye images.

### Deep learning model architecture

**Fig. 1:**
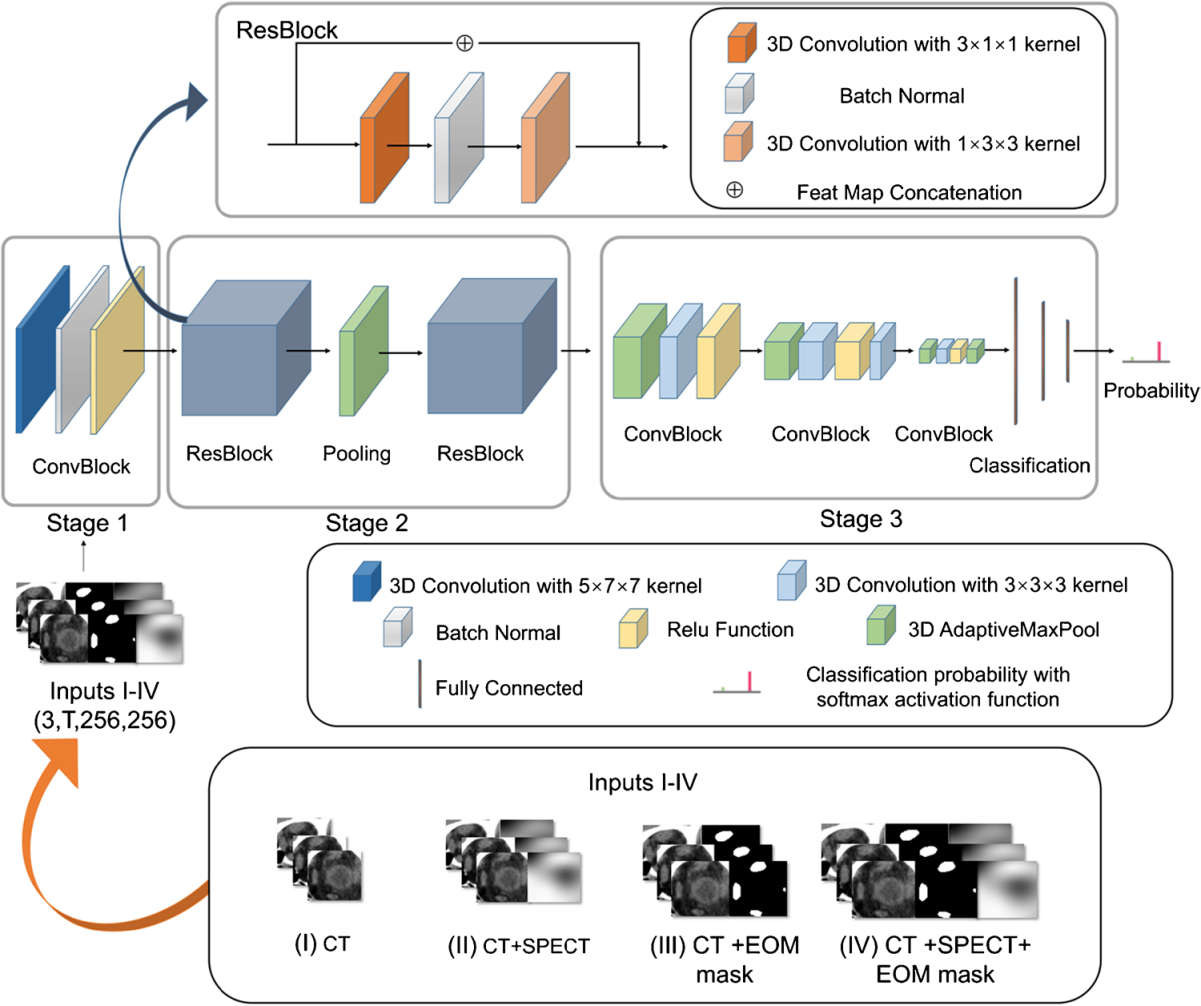
Flowchart of the classification network in the GO-Net architecture. GO-Net’s classification stage consists of three parts: the first part includes a 3D ConvBlock layer; the second part consists of two 3D ResBlock layers and a pooling layer; and the third part consists of three ConvBlock layers and a classification layer

To automatically identify active GO patients, we developed a deep learning-based approach called GO-Net. GO-Net includes a SV-Net for extracting EOMs and a 3D convolutional neural network (CNN) for classifying active and inactive GO. The details of the model architectures in the GO-Net segmentation stage are presented in Supplementary Fig. 2. For the GO-Net classification stage, inspired by the study of Wang et al. [20], we employed a combination of SPECT/CT images and the EOM masks, derived from the segmentation results, to construct multi-channel image, which was utilized as input to the GO classification network, thus generating four models with different inputs as follows: (i) the model employs only CT images as a single-channel input. (ii) The model utilizes a dual-channel input, composed of both CT and SPECT images. (iii) The model utilizes a dual-channel input, composed of both CT and EOM masks. (iv) The model employs a three-channel input, consisting of CT and SPECT images along with EOM masks. The architecture of the GO-Net classification network is shown in Fig. 1.

The above analyses were implemented in Python using the PyTorch library, and the model was trained on a Tesla V100 GPU with 32 GB GPU memory. In terms of parameters, both models were trained by using the adaptive moment estimation optimizer with a learning rate of 0.0001. The batch sizes of the segmentation and classification models were set to 4 and 1, respectively, and the number of training epochs was set to 125 and 100, respectively.

### Evaluation

To evaluate the performance of the segmentation model, the intersection over union (IOU), specificity (SP), and sensitivity (SN) (Equations 2–4) were calculated on the training and validation sets, respectively.

2 $$IOU\left({R}_{k},{R}_{k}^{^{\prime}}\right)=\frac{\left|{R}_{k}\cap {R}_{k}^{^{\prime}}\right|}{\left|{R}_{k}|\cup {|R}_{k}^{^{\prime}}\right|}$$

3 $${\text{SP}}\left({R}_{k},{R}_{k}^{^{\prime}}\right)=\frac{\left|{R}_{k}\cap {R}_{k}^{^{\prime}}\right|}{\left|{R}_{k}^{^{\prime}}\right|}\times 100\%$$

4 $$SN\left({R}_{k},{R}_{k}^{^{\prime}}\right)=\frac{\left|{R}_{k}\cap {R}_{k}^{^{\prime}}\right|}{\left|{R}_{k}\right|}\times 100\%$$

In Equations (2–4), *R*_*k*_ is the _k_th predicted image after postprocessing, and *R'*_*k*_ is the _*k*_th ground truth image.

To assess the performance of the classification models, receiver operator characteristic (ROC) curves were plotted for the test set. The accuracy, precision, sensitivity, specificity, F1 score, and AUC were also calculated.

To better visualize the effect of the GO-Net model, a gradient weighted class activation map (Grad-CAM) was used to identify and highlight the different areas during the classification process.

## Results

### Segmentation performance

The GO-Net segmentation model was trained on the training set (155 eyes), which required approximately 165 min, while predicting the EOM segmentation results on the validation set took an average of 4.7 s per eye. The performance of the segmentation model for each rectus muscle in the validation set is shown in Table 1. Overall, the IOU, SN, and SP of the average rectus muscle were 0.82 ± 0.05%, 91.01 ± 5.32%, and 99.96 ± 0.03%, respectively. As illustrated in Supplementary Fig. 3, the EOM masks predicted by our segmentation model approximately overlap with the ground truth results, indicating the good performance of the model. Nevertheless, the segmentation results of 47 eyes did not match the actual EOMs and were adjusted manually (Supplementary Fig. 4), which took an average of five minutes per eye.

**Table 1:** Performance of the segmentation models on the validation set ($n=\overline{x }\pm s$)

	SR	LR	MR	IR	Average
IOU	0.79 ± 0.03	0.81 ± 0.06	0.85 ± 0.08	0.82 ± 0.08	0.82 ± 0.05
SP (%)	99.93 ± 0.04	99.97 ± 0.02	99.96 ± 0.03	99.96 ± 0.03	99.96 ± 0.03
SN (%)	89.13 ± 3.92	90.81 ± 4.71	92.35 ± 7.52	90.62 ± 7.93	91.01 ± 5.32

*SR* superior rectus muscle, *LR* later rectus muscle *MR* medial rectus muscle, *IR* inferior rectus muscle

### Classification performance

**Fig. 2:**
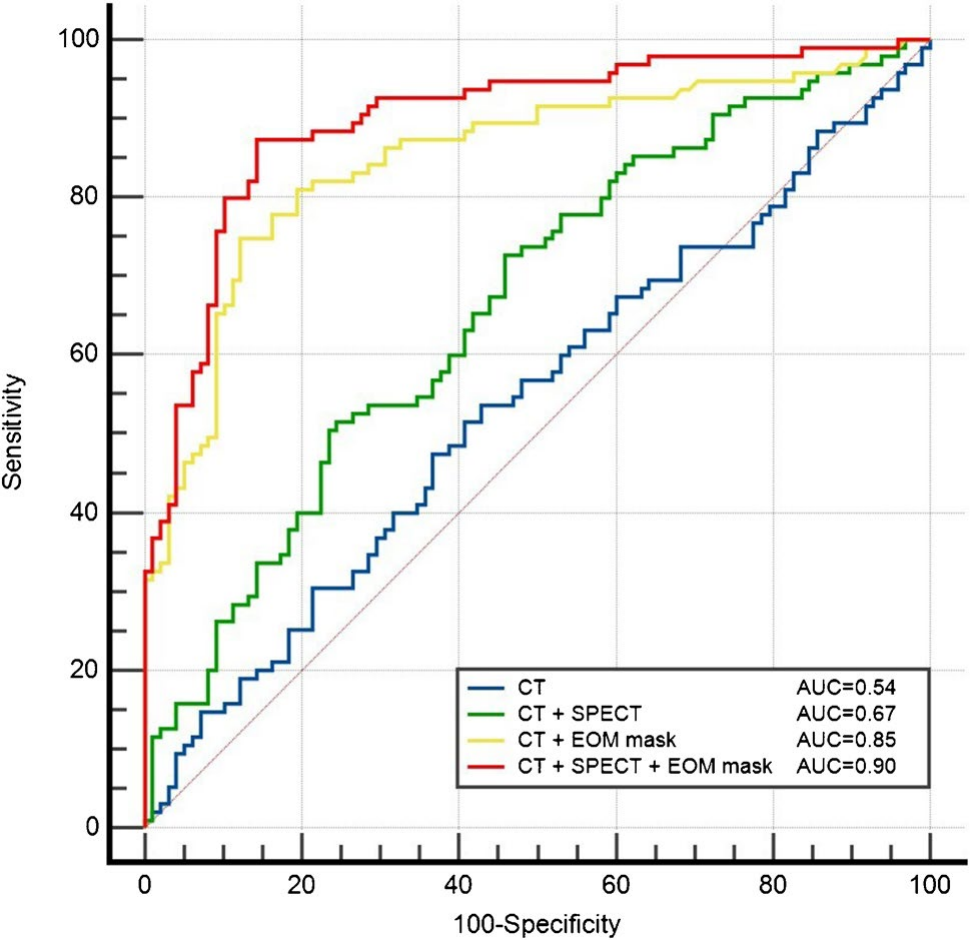
ROC curves of the classification models with different inputs on the test set

The GO-Net classification model was trained on the training set (765 eyes) in approximately 42 min, while on the test set, the classification of GO activity took approximately 1.23 s per eye on average. First, we compared the GO-Net classification models with three different inputs. As expected, the best performance on the test set was obtained by the three-channel input model combining CT, SPECT, and EOM masks, reaching an AUC score of 0.90 and a diagnostic accuracy of 86.10% for predicting active GO. The two-channel input model, which combined CT and SPECT data, achieved an AUC score of 0.67 and a diagnostic accuracy of 60.88%. The single-channel input model, which only used CT images, had the lowest predictive value with an AUC score of 0.54. Four different GO-Net classification models with distinct inputs were evaluated through fivefold cross-validation to determine the optimal model for each input. The ROC curves and precision, sensitivity, specificity, and F1 scores are shown in Fig. 2 and Table 2. Table 3 shows the metrics for the best GO-Net classification model, and the AUCs were 0.90 ± 0.01, 0.89 ± 0.01, and 0.89 ± 0.02 on the training, validation, and test sets, respectively. Furthermore, given the heterogeneity of GO among patients with different ages and sexes, we compared the model performance in different sex and age subgroups on the test set (Supplementary Table 1). The classification model obtained better performance for males than females (AUC: 0.94 vs. 0.85), while the model showed similar performance for older and younger patients (AUC: 0.86 vs. 0.89).

**Table 2:** Performance of the classification models with different inputs on the test set

	Accuracy(%)	Precision (%)	Sensitivity (%)	Specificity (%)	F1 score	AUC
CT	54.92	55.88	57.14	53.68	0.47	0.54
CT + SPECT	60.88	58.56	51.58	75.51	0.63	0.67
CT + EOM mask	75.13	77.65	74.74	87.76	0.81	0.85
CT + SPECT + EOM masks	86.10	85.17	87.37	85.71	0.86	0.90

**Table 3:** Performance of the three-channel input model on the training, validation and test sets ($n=\overline{x }\pm s$)

	Accuracy (%)	Precision (%)	Sensitivity (%)	Specificity (%)	F1 score	AUC
Training set	84.31 ± 0.25	85.67 ± 1.06	86.64 ± 0.42	82.32 ± 1.28	0.85 ± 0.01	0.90 ± 0.01
Validation set	82.06 ± 0.32	85.01 ± 2.35	83.63 ± 1.54	82.53 ± 1.26	0.83 ± 0.02	0.89 ± 0.01
Test set	84.25 ± 1.25	83.35 ± 1.53	84.63 ± 0.84	83.87 ± 1.97	0.83 ± 0.01	0.89 ± 0.02

### Visualization

**Fig. 3:**
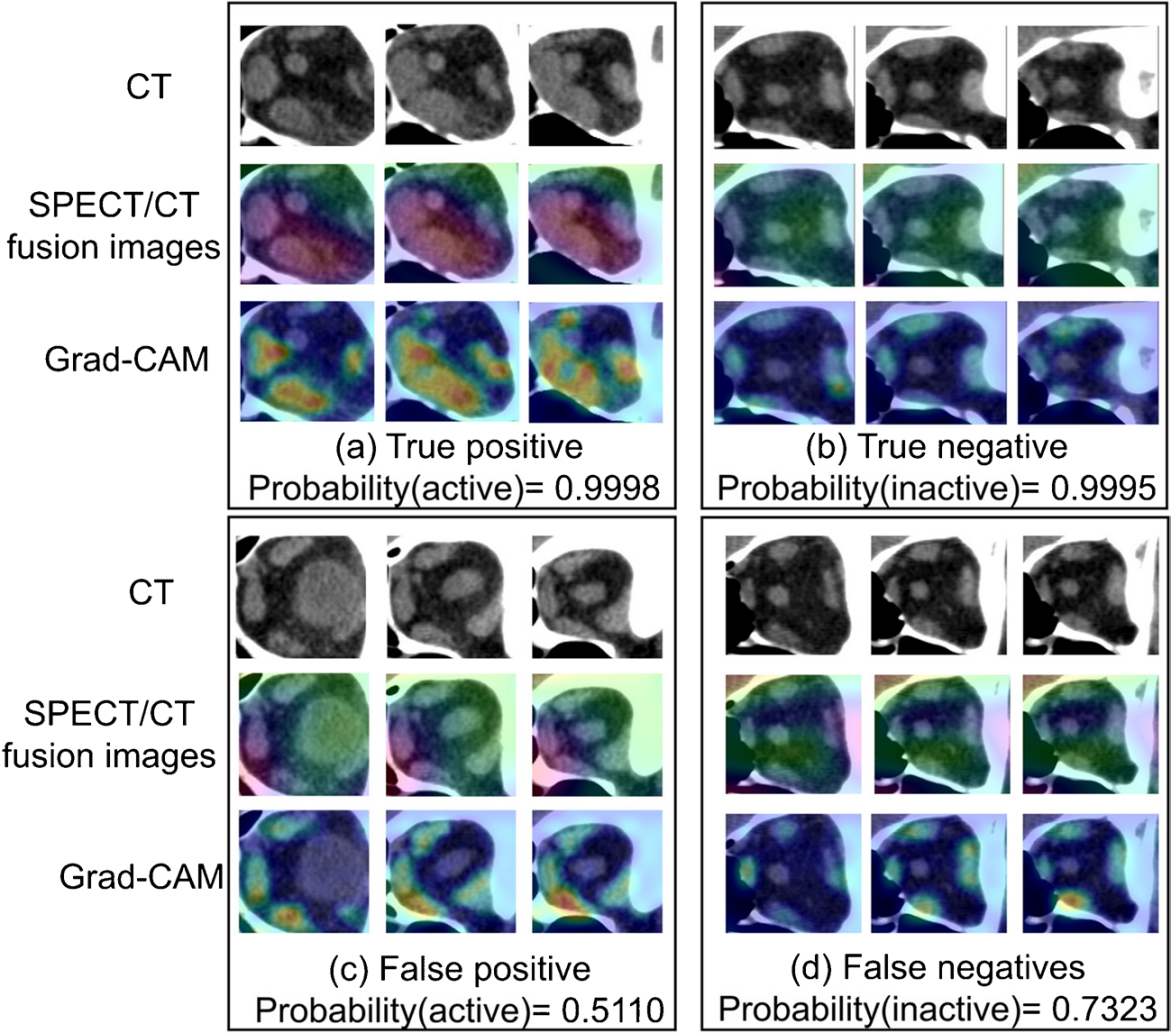
Grad-CAM results of the GO-Net classification model. In order to enhance the comprehension of the GO-Net classification model’s decision-making process, four illustrative eyes (left eyes belonging to patients with GO) were randomly selected, and the orbital images (CT scans, SPECT/CT fusion images, and Grad-CAM results with CT scans as a base) of their coronal surfaces centered around the optic nerve were displayed. The superior rectus and inferior rectus correspond to the upper and lower sides of the patient’s image, while the internal rectus and external rectus correspond to the left and right sides, respectively. (a) True positive: clinical diagnosis of activity and model classification result of activity; (b) true negative: clinical diagnosis of inactivity and model classification result of inactivity; (c) false positive: clinical diagnosis of inactivity and model classification result of activity; (d) false negative: clinical diagnosis of activity and model classification result of inactivity. Probability (active): probability that the sample is in the active phase; probability (inactive): probability that the sample is in the inactive phase

According to Grad-CAM analyses, our classification model could automatically identify thickened or DPTA-avid EOMs. Example images of accurate predictions (true positives and true negatives) and false predictions (false positives and false negatives) are shown in Fig. 3. Furthermore, to visualize the role of EOMs in diagnosing active GO, we randomly selected 3 left eyes with active GO and 3 left eyes with inactive GO. As shown in a schematic 3D segmentation of the CT and SPECT EOMs (Supplemental Fig. 5), the EOMs in the active GO group were thicker and less attenuated than those in the inactive GO group, which is consistent with our previous orbital SPECT/CT findings [7].

## Discussion

In this study, we developed a two-stage deep learning method based on orbital [^99m^Tc]TcDTPA SPECT/CT images that was highly accurate in distinguishing the active and inactive phases of GO (AUC = 0.89). Furthermore, our segmentation module extracted the EOMs in orbital CT images well (IOU = 0.82).

### [^99m^Tc]TcDTPA SPECT/CT imaging for classification of GO

Several previous studies have demonstrated that orbital [^99m^Tc]TcDTPA SPECT/CT can be used to obtain accurate assessments of disease activity in patients with GO. Szumowski et al. [6] concluded that SPECT/CT had a high sensitivity of 93% and a specificity of 89% for diagnosing GO. We previously reported that the DPTA uptake ratio (UR) within EOMs was correlated with the CAS (*R* = 0.77, *P* < 0.01) [21]. However, the semiquantitative UR measurements varied depending on the choice of referenced denominators, which may reduce the reproducibility of the results. On the other hand, in the active stage of GO, infiltration with inflammatory cells and increased proteoglycan content may cause edematous swelling of orbital soft tissues and enlarged EOMs. Therefore, EOM involvement is a critical feature of GO. The EOM volume has been proven to be a reliable parameter for judging the staging and therapeutic efficacy in patients with GO [22]. However, these measurements are difficult and require computer analyses, including specific application software and hardware, as well as considerable radiologist time [21]. Additionally, EOM enlargement can occur in different phases, and it may be challenging to stage GO using only morphologic parameters. Thus, a fully automatic method for assessing EOMs by combining morphological and functional features using hybrid SPECT/CT is highly desirable.

### Machine learning for GO diagnosis and activity assessment

Several studies have demonstrated that ML can be applied to automatically screen and diagnose various ocular disorders, such as cataracts, diabetic retinopathy, glaucoma, age-related macular degeneration, and premature retinopathy [8]. Nevertheless, ML-based techniques using orbital imaging have rarely been investigated in diagnosing GO and assessing GO activity. Hu et al. [9] used an ML model to classify GO using MRI. They considered a group of 60 patients with active GO and 40 patients with inactive GO. They determined variations in the magnetization transfer ratio, signal intensity ratio (SIR), and apparent diffusion coefficient (ADC) of the EOMs for each eye according to the MRI images. Their ML-based model obtained better performance for disease activity differentiation and CAS prediction than a model that combined SIRs and ADCs (AUC, 0.93 vs. 0.90). However, the study cohort was small, and only preplanned features were used in the proposed ML model. Song et al. [15] built a deep learning model for screening GO using 784 (normal: 625, GO: 168) orbital CT images to train the model and 114 and 227 orbital CT images as the validation and test sets, respectively. This study achieved good results (accuracy, 87%; sensitivity, 88%; specificity, 85%). However, the sample of GO patients was small, and GO activity was not staged. Chen et al. [10] proposed an algorithm based on a deep convolutional neural network (DCNN) for detecting GO activity. This algorithm was trained using 160 orbital MRI images of GO patients (50 active, 110 inactive), with 80% of the images used for training and validation and the remaining 20% used for testing. The accuracy, precision, sensitivity, specificity, and F1 score of the resulting best model were 85.5%, 64.0%, 82.1%, 86.5%, and 0.72, respectively. However, this study included only 32 GO patients (7 active, 25 inactive) in the test set, which was unbalanced, resulting in overfitting of the model.

### Strengths of our study

Our study proposed a deep learning-based method using SPECT/CT images. Compared to previous studies using ML methods to assess GO, our model utilized both anatomic and functional features. Moreover, our method has two important modules: EOM segmentation and disease activity classification. The EOM mask segmentation results were used as inputs to the classification network. Compared with our previously developed algorithm [14], the IOU on the superior rectus muscle increased from 0.74 to 0.79.

In terms of classification modules, the three-channel input models comprising SPECT, CT, and EOM masks achieved the highest accuracy of 86.10%. In contrast, the accuracy of the two-channel model incorporating SPECT and CT was 60.88%, while the accuracy of the single-channel model using only CT images was the lowest at 54.92%. Furthermore, the findings highlighted that the utilization of the three-channel architecture method could significantly improve the diagnostic sensitivity and specificity of GO activity staging. Moreover, this three-channel input model achieved a higher sensitivity, precision, and F1 score than the DCNN and MRI-based architecture developed by Chen et al. [10], with sensitivities, precisions, and F1 scores of 84.6% vs. 82.1%, 83.4% vs. 64.0%, and 0.83 vs. 0.72, respectively.

It is worth noting that we used a new criterion that combined the CAS with SPECT images to annotate the activity stage, in contrast to previous ML studies, which used only on the CAS [9, 10, 15]. We initially constructed a CAS-based classification model. However, its accuracies on the training, validation and test sets were substantially lower than those of the model based on the combined criteria (see Supplementary Methods and Table 2), mainly due to the subjective nature of the CAS, which may underestimate the inflammatory activity in certain patients [5, 23].

Moreover, the GO-Net model has good interpretability. The true positive case shown in Fig. 3a illustrates a strong agreement between the model’s focus area and the area with thickened EOMs and increased DTPA uptake. In particular, the inferior and medial rectus have been investigated more attention than other recti. In the true negative cases [Fig. 3b], the classification model has the same attention for the four EOMs, which is consistent with the negative results of the SPECT/CT images. These results indicated that GO-Net could automatically focus on the pathological changes in the four EOMs and provide different levels of attention during staging. Notably, the false positive case shown in Fig. 3c was observed in a region in the adjacent nasal sinuses with avid DPTA uptake. However, the patient was not clinically rated as inactive, and the judgment of the model was wrong due to the inflammation caused by sinusitis (Supplementary Fig. 6). Moreover, we found that CAS and SPECT/CT findings were not always consistent, as shown in Fig. 3d: according to the CAS (CAS = 4), this case should be diagnosed as active GO, while no significant morphological or functional changes were found in the SPECT/CT images, resulting in a negative result. Although previous studies have shown a significant positive correlation between the CAS and DTPA uptake [5], some individuals with CASs > 3 had low DTPA uptake and did not respond favorably to anti-inflammatory treatment [24]. In line with these results, the Grad-CAM derived from our model failed to identify suspicious lesions, further indicating that the CAS has limitations for GO staging.

### Limitations

This work has several limitations. First, our patient population was from a single medical center. Although SPECT/CT enables more accurate and objective staging of GO activity than other approaches, we were unable to obtain sufficiently large samples from other medical centers due to cost and technical issues, which may lead to selection bias. Nevertheless, this technology is being utilized by an increasing number of nuclear medicine laboratories, and we hope to validate this model with multicenter cohorts in the future. Second, the proposed model predicts the diagnosis based on only SPECT/CT scans. In practice, clinicians combine other clinical assessments and follow-up data to make the final decision. We believe that our model can achieve better results if other clinical assessments are added to the model training process. Thirdly, since the number of patients who underwent SPECT/CT pre- and post-treatment is too small, future studies will prioritize a larger cohort with comprehensive treatment data to validate the model's predictive efficacy for treatment response. In addition, this study is limited to staging the activity of GO, and diagnostic and prognostic results should be combined to build an improved intelligent medical system.

## Conclusions

We propose a deep learning-based approach known as GO-Net that automatically detects GO activity by using SPECT/CT images. The GO-Net-assisted strategy could effectively differentiate active and inactive GO, thus potentially improving the efficiency and reproducibility of GO staging in clinical settings.

### Supplementary Information

Below is the link to the electronic supplementary material.

Supplementary file1 (DOCX 9.93 MB)

## Data Availability

The SPECT/CT images used in this study were obtained from Xiangya Hospital and were restricted by the Ethics Committee of Xiangya Hospital to protect patient privacy. Further inquiries can be directed to the corresponding author.
