# Supplementary material for: Deep learning-based diagnosis of disease activity in patients with Graves’ orbitopathy using orbital SPECT/CT
Source: Eur J Nucl Med Mol Imaging. 2023 Jul 3;50(12):3666–74. doi: 10.1007/s00259-023-06312-2 (PMC10547836; doi:10.1007/s00259-023-06312-2)
Supplement: Supplementary file 1 — Supplementary file1 (DOCX 9.93 MB) [file 259_2023_6312_MOESM1_ESM.docx]

Deep Learning-Based Diagnosis of Disease Activity in Patients with Graves’ Orbitopathy Using Orbital SPECT/CT

Ni Yao^1^, Longxi Li^1^, Zhengyuan Gao^2^, Chen Zhao^3^, Yanting Li^1^, Chuang Han^1^, Jiaofen Nan^1^, Zelin Zhu^1^, Yi Xiao^4^, Fubao Zhu^1^, Min Zhao^5*^, Weihua Zhou^3,6^

^1^School of Computer and Communication Engineering, Zhengzhou University of Light Industry, Zhengzhou, Henan, 450002, China.

^2^School of Biomedical Engineering, Shenzhen University Medical School, Shenzhen University, Shenzhen, 518060, China

^3^Department of Applied Computing, Michigan Technological University, Houghton, MI, USA.

^4^Department of Nuclear Medicine, Xiangya Hospital, Central South University, Changsha, China.

^5^Department of Nuclear Medicine, The Third Xiangya Hospital, Central South University, Changsha, China.

^6^Center for Biocomputing and Digital Health, Institute of Computing and Cybersystems, and Health Research Institute, Michigan Technological University, Houghton, MI, USA.

*Corresponding Authors:

Min Zhao, M.D, Ph.D

Department of Nuclear Medicine, The Third Xiangya Hospital of Central South University

No. 138, Tongzipo Road, Changsha, Hunan Province, China, 410013

**Supplementary Methods**

In the initial phase of this study, the CAS was used as the sole diagnostic criterion for GO activity. The criteria were set as follows: (1) eyes with CASs≥3/7 were labeled as active; (2) eyes with CASs<3/7 were labeled as inactive. After labeling, 287 eyes were labeled active, and 669 eyes were labeled inactive. A random split of 20% of the total dataset was used to create the test dataset, and the remaining 80% of the data were separated into training and validation sets (4:1 ratio) to perform fivefold cross-validation.

In the GO-Net model training process, during the classification stage, the three-channel image composed of CT, SPECT and EOM masks was used as the input to the classification model. In terms of parameters, the model was trained by an adaptive moment estimation optimizer with a learning rate of 0.0001. The batch size of the model was set to 1. The number of training rounds was set to 100.

The performance of the classification model based on only the CAS is shown in **Supplementary Table 2**. The classification accuracy of the model based on only the CAS is worse than that of the model using a combination of CAS and SPECT images, mainly due to the subjective nature of the CAS, which seriously underestimates the inflammatory disease activity in some patients.

**Supplemental Table**

**Supplemental Table 1 Performance of the classification model on the test set grouped by sex and age (n=**$\bar{\boldsymbol{x}}$**±s).**

|  | Accuracy  (%) | Precision  (%) | Sensitivity  (%) | Specificity  (%) | F1 score | AUC |
| --- | --- | --- | --- | --- | --- | --- |
| Male(n=75) | 89.67±1.46 | 87.32±1.81 | 92.61±1.07 | 86.81±2.08 | 0.89±0.01 | 0.94±0.01 |
| Female(n=118) | 79.6±0.80 | 78.27±3.78 | 81.63±2.91 | 77.65±1.63 | 0.80±0.01 | 0.85±0.01 |
| Age < 45(n=99) | 83.03±1.34 | 78.55±2.29 | 76.84±2.00 | 86.89±1.80 | 0.78±0.02 | 0.86±0.01 |
| Age >= 45(n=94) | 84.89±1.24 | 87.97±1.51 | 87.02±1.79 | 81.62±2.65 | 0.87±0.01 | 0.90±0.01 |

**Supplemental Table 2 Performance of the classification model based on the CAS (n=**$\bar{\boldsymbol{x}}$**±s).**

|  | Accuracy  (%) | Precision  (%) | Sensitivity  (%) | Specificity  (%) | F1 score | AUC |
| --- | --- | --- | --- | --- | --- | --- |
| Training set | 76.05±1.17 | 79.08±1.66 | 89.43±3.84 | 68.56±5.22 | 0.83±0.01 | 0.75±0.06 |
| Validation set | 75.15±2.14 | 79.23±1.73 | 85.23±2.62 | 67.65±1.63 | 0.82±0.01 | 0.75±0.07 |
| Test set | 77.20±1.24 | 80.09±0.08 | 89.62±3.52 | 68.41±3.75 | 0.90±0.03 | 0.78±0.04 |

**Supplemental Figure legends**

**Supplemental Figure 1 Binarization of** **EOM Ground Truth.**


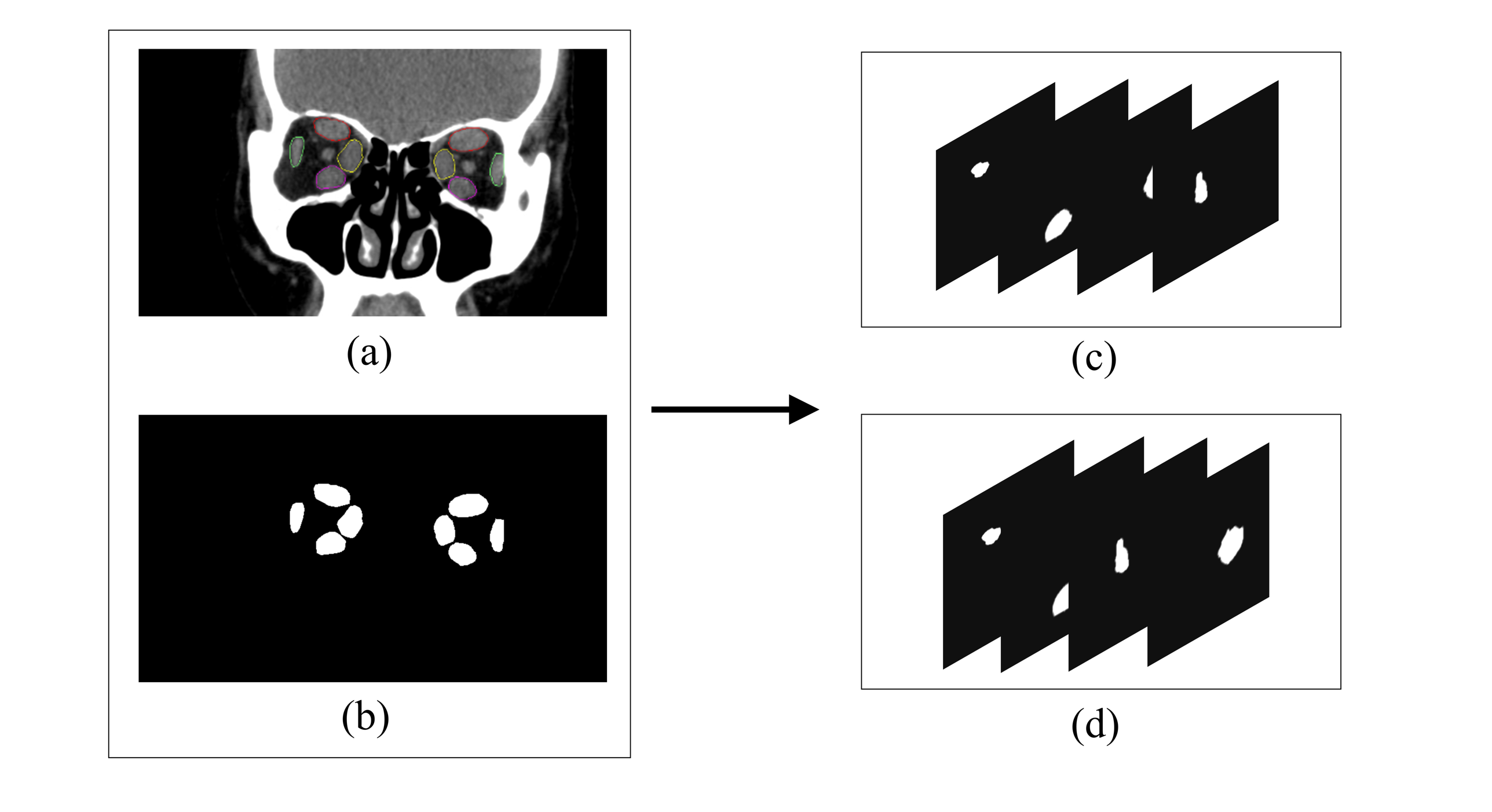


(a) EOM ground truth in CT images. (b) A binary image of the EOM was generated based on its ROI. (c) Binary maps were generated for each individual EOM of the left eye. (d) Binary maps were generated for each individual EOM of the right eye.

**Supplemental Figure 2 Flowchart of the GO-Net segmentation network.**





**Supplemental Figure 3** **Semantic segmentation results.**


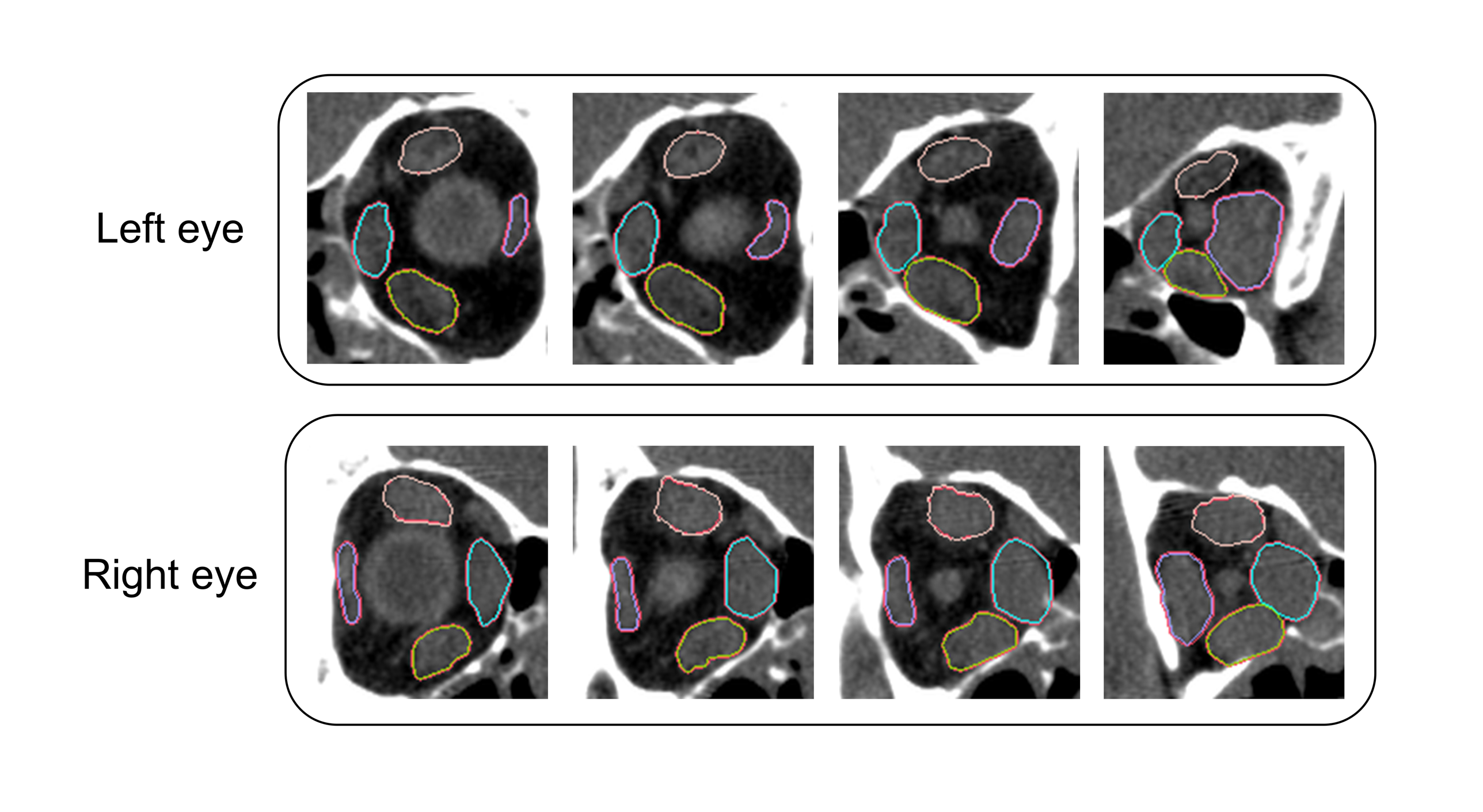


The ground truth is presented as red ROI. The colors in the annotations reflect the ROI of the segmentation results: blue, medial rectus muscle; white, superior rectus muscle; purple, later rectus muscle; yellow, inferior rectus muscle.

**Supplemental Figure 4 Illustration of manual correction.**


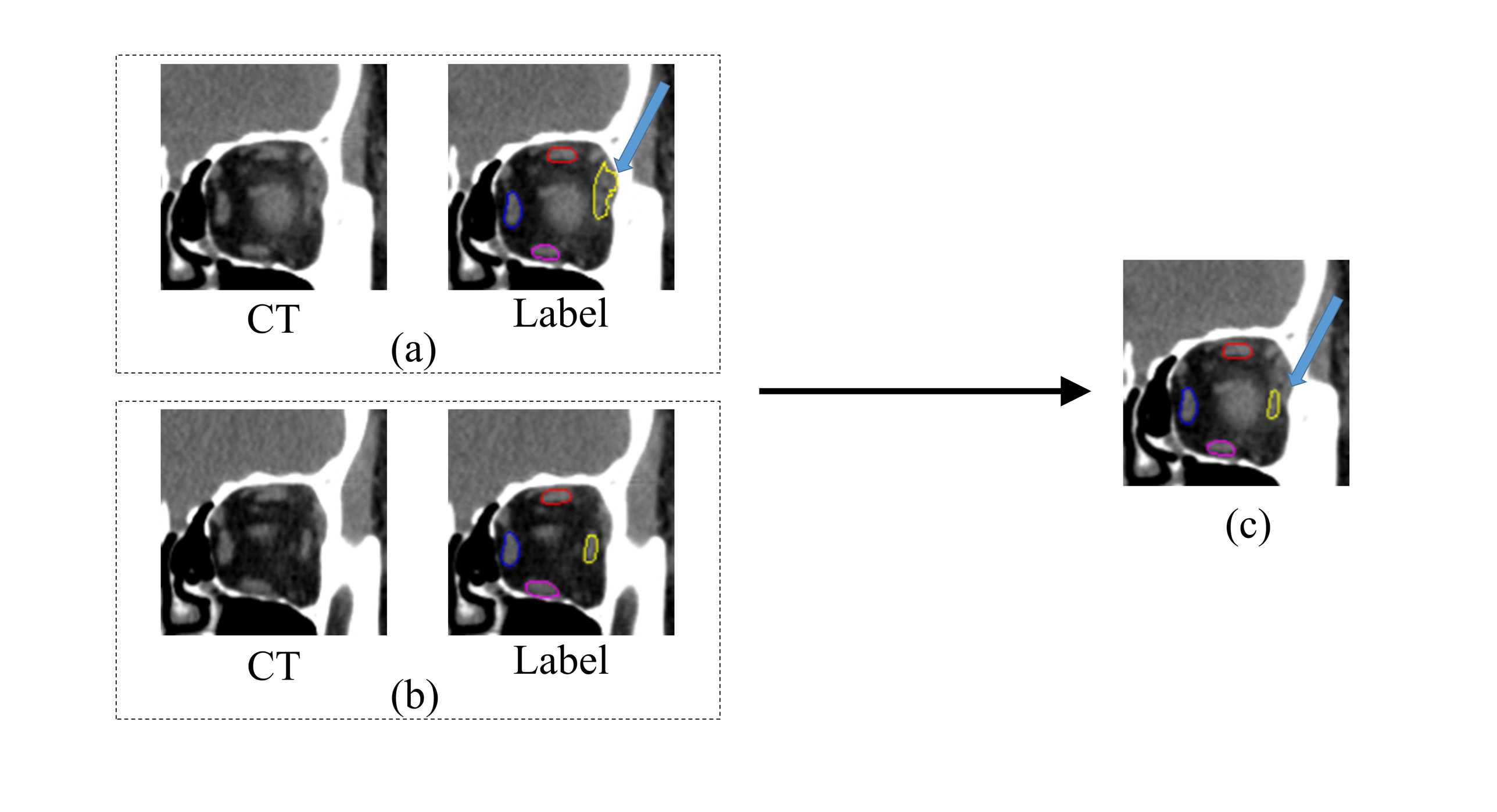


The blue arrows highlight the segmentation results requiring manual modification. (a) and (b) represent the anterior and posterior sections of the patients' CT images. Based on the separation of the external ocular muscle and fat in (b), it is evident that the predictions in (a) are flawed. The segmentation model erroneously classified the fat within the orbit as part of the external ocular muscle. This misclassification highlights the incorrectness of the initial predictions. The corrected outcome is depicted in (c).

**Supplemental Figure 5 Three-dimensional morphology and function of EOMs derived from orbital SPECT/CT images.**


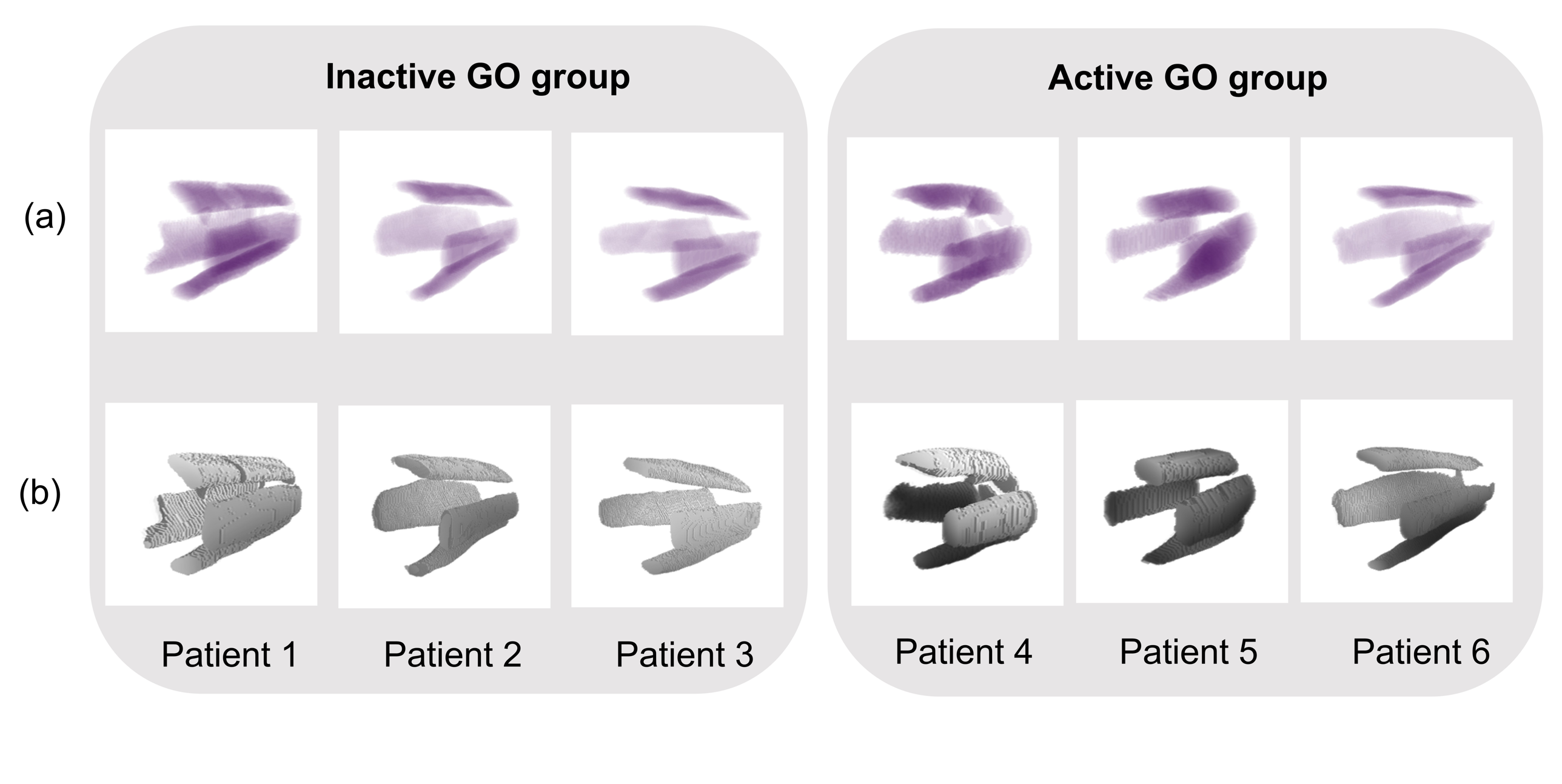


(a) 3D morphology of EOMs derived from CT; (b) DTPA uptake of EOMs derived from SPECT.

**Supplemental Figure 6 Coronal views of SPECT/CT fusion images.**





The red box marks the eye of interest in a patient who experienced high DTPA uptake in the adjacent sinuses, leading to the misinterpretation of their medial rectus and inferior rectus muscles by the model as areas where lesions occurred. (a), (b), (c), (d): different slices of SPECT/CT fusion images.
